# Intestinal Ligation Mimicking Ureteral Ligation After Ovariohysterectomy in an 11-Month-Old Chihuahua

**DOI:** 10.1155/2024/8550288

**Published:** 2024-10-03

**Authors:** Corey J. Fisher, Cristina Piedrahita, Olivia Choe, Amanda James, Logan M. Scheuermann, Elodie Huguet, Carl Southern

**Affiliations:** Department of Small Animal Clinical Sciences, Veterinary Teaching Hospital, University of Florida, 2015 S.W. 16th Ave, Gainesville, Florida 32608, USA

## Abstract

Intestinal ligation during ovariohysterectomy has not been previously reported in dogs. Risk factors for this complication appear to be the same as those for ureteral injury, namely decreased surgical visualization due to small patient size and small incision size. This case report describes how the presentation of intestinal ligation in a 2-kg, 11-month-old chihuahua can mimic that of iatrogenic ureteral injury with vomiting, anorexia, and severe azotemia. Ultrasound served as a key diagnostic to visualize intestinal mechanical obstruction, an encircling ligature around a segment of jejunum with no blood flow on Doppler interrogation, and normal appearance and blood flow of the ureters and kidneys. The treatment consisted of aggressive fluid therapy, circulatory support, and emergent resection and anastomosis of the necrotic portion of the intestines. Within 48 h, the dog's azotemia was resolved, and it was reported to be doing well at 1-month follow-up.

## 1. Introduction

Ovariohysterectomy (OVH) is the most commonly performed abdominal surgery in small animal medicine in the United States due to its utility in controlling pet populations, preventing unwanted sexual behaviors, and reducing the risk for pyometra and mammary cancer [1, 2]. Numerous complications from this routine surgery have been described previously, and the incidence of complications can be as high as 20% [1–3]. The most immediate life-threatening complications of OVH are hemorrhage, which is more common in larger dogs, and ureteral damage, which is more common in small dogs and cats [4, 5]. Ureteral damage commonly presents as lethargy, vomiting, anorexia, and severe azotemia [5].

Accidental intestinal entrapment during OVH is an equally life-threatening complication but exceedingly rare with only two related case reports. The first occurred in a 5-month-old cat in which its colon was entrapped along with a circumferential ligature around the uterine horns [6]. Similarly, another cat developed intestinal strangulation post-OVH due to entrapment in a mesenteric rent [7]. Both cats were treated successfully with a resection and anastomosis of the ischemic portion of the intestines. Similar to these reports of intestinal entrapment, the current veterinary literature describing ureteral injury implicates small size of both the patient and the surgical incision as risk factors for iatrogenic visceral injury during routine OVH [5–7].

The aim of this case report is to describe how the clinical signs and biochemical abnormalities associated with intestinal ligation post-OVH in an 11-month-old chihuahua mimicked that of ureteral injury, how diagnostic imaging was key in differentiating these conditions, and how rapid surgical management led to complete recovery and a positive outcome long term.

## 2. Case Report

An 11-month-old spayed female chihuahua weighing 2.1 kg was referred for evaluation of vomiting, anorexia, and lethargy 2 days after OVH. The dog was reported to be healthy prior, and the procedure, including anesthesia, was noted to be routine. The dog was discharged the same day as surgery and given one dose of meloxicam (0.1 mg/kg orally) that evening, after which it started vomiting. Over the next 2 days, the dog continued to vomit and developed anorexia, lethargy, and abdominal pain. She had no bowel movements but was reportedly urinating normally.

The dog re-presented to the referring veterinarian on Day 2 postoperatively, where she was 10% dehydrated, mildly tachycardiac (130 bpm), and mildly hypothermic (98°F). Serum biochemistry revealed severe azotemia (creatinine too high to read, ref. 0.5–1.8 mg/dL; BUN 69, ref. 7–27mg/dL), hyperphosphatemia (> 16.1, ref. 2.5–6.8 mg/dL), hypochloremia (95, ref. 109–122mmol/L), and elevated ALP (542, ref. 23–212 U/L). Complete blood count revealed lymphocytosis (5.43K/*μ*L, ref. 1.05–5.10K/*μ*L) and monocytosis (3.70K/*μ*L, ref. 0.16–1.12K/*μ*L) with a band neutrophilia suspected. The values were reported from point of care analyzers and not confirmed by a blood film review. A single lateral abdominal radiograph reportedly revealed gas dilated loops of the small intestine with no appreciable foreign material. The dog was treated with IV fluids (lactated Ringer's solution (LRS)) at a maintenance fluid rate (50 mL/kg/day) plus correction of 10% dehydration over 24 h, maropitant (1 mg/kg IV), ondansetron (0.2 mg/kg IV), famotidine (1 mg/kg IV), ampicillin-sulbactam (22 mg/kg IV), and buprenorphine (0.01 mg/kg SQ). Due to failure to improve after 6 h, the dog was referred to a university teaching hospital.

On presentation, the dog was dull, 7% dehydrated, mildly tachycardic (128 bpm), and hypotensive (70 mmHg with a Doppler). Her rectal temperature was 99.3°F. Initial venous blood gas confirmed a severe azotemia (creatinine too high to read, ref. 0.8–1.5 mg/dL), while point-of-care ultrasound revealed a subjectively underloaded left ventricle and severe fluid dilation of the stomach and several loops of the intestine. A moderately distended urinary bladder was visualized, and urinalysis (via cystocentesis) revealed a urine specific gravity of 1.020 (ref. 1.012–1.050).

**Figure 1:**
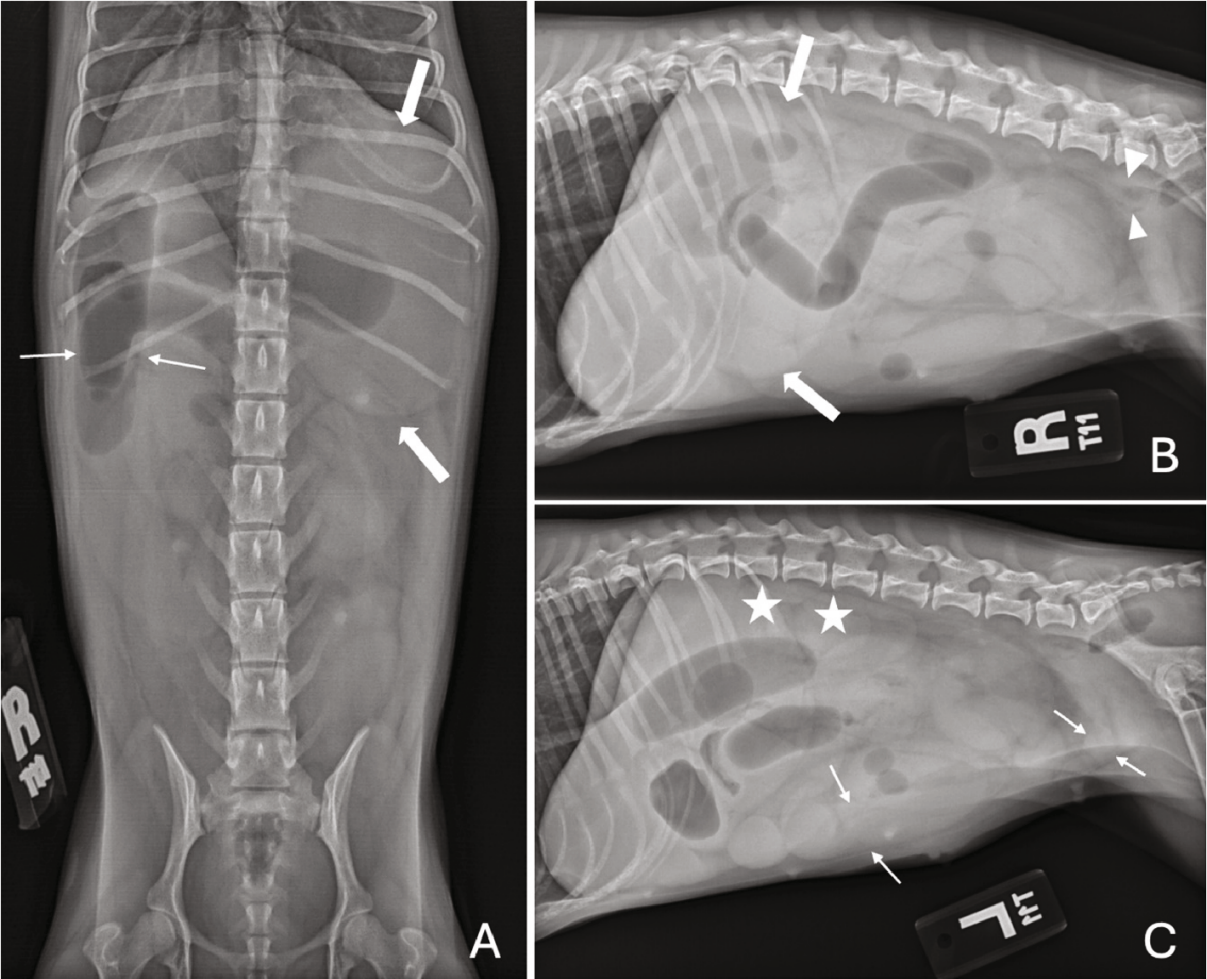
Ventrodorsal (A), right lateral (B), and left lateral (C) radiographic abdominal projections of an 11-month-old, female castrated chihuahua referred for evaluation of severe azotemia postovariohysterectomy. There is severe gastric (bold arrows) and segmental small intestinal dilation (nonbold arrows) indicative of mechanical obstruction. The cause is not apparent. The colon is empty (arrowheads). Note that the renal silhouettes (stars) are normal.

**Figure 2:**
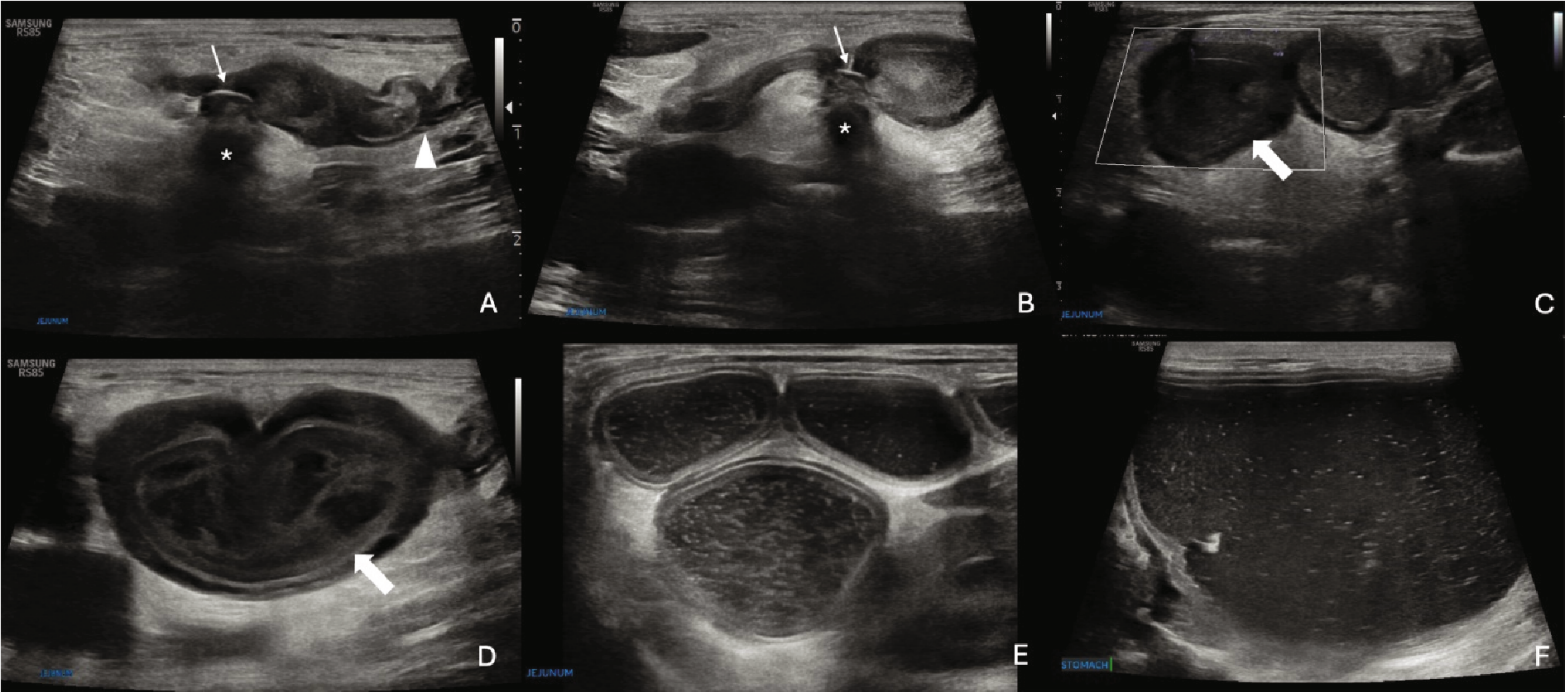
(A–F) Ultrasonographic still images of the gastrointestinal tract of the same patient in Figure 1. Hyperechoic, well-defined suture material (nonbold arrows) encircles a folded and severely dilated jejunal segment (bold arrows) forming a hairpin loop in the region of the left ovarian pedicle (asterisk). The wall of the affected small intestinal segment is moderately thickened with maintenance of wall layer distinction and lack of blood flow on microvascular Doppler interrogation (C, D), consistent with intestinal necrosis. Marked focal steatitis and mild anechoic peritoneal effusion surround the affected jejunal segment. Orad, the stomach (F) and small intestine (E) are markedly dilated, consistent with mechanical obstruction. Aborad, the small intestine is normal and collapsed (arrowhead), supporting the presence of two population of bowel. Cineloop of a ligated segment of small intestinal is available in the supplemental materials.

The dog was immediately treated with a 20 mL/kg IV LRS bolus over 15 min, and blood pressure subsequently improved to 95 mmHg. Abdominal radiographs confirmed the presence of gastric and segmental small intestinal gas and fluid distension with no overt intraluminal foreign objects observed (Figure 1). The caudal vena cava appeared small further supporting the suspected hypovolemia. Abdominal ultrasound was then performed and confirmed severe fluid distension of the stomach and small intestine to the level of the mid-jejunum where a jejunal segment made a sharp hairpin turn in the region of the left ovarian pedicle (Figure 2 and Figure S1). At the base of the loop, the intestine was focally constricted with encircling hyperechoic suture material. The wall of the affected jejunal segment was thickened with maintained wall layer distinction and contained no apparent blood flow on color and power Doppler interrogation. The surrounding mesenteric fat was hyperechoic and dissected by a small amount of anechoic effusion. Aborad to this, the remainder of the small intestine was empty, supporting the radiographic finding of two populations of the small intestine. Both kidneys and ureters were normal without evidence of ureteral ligation, and appropriate blood flow was noted on Doppler interrogation. These findings confirmed the presence of mechanical obstruction of the small intestine secondary to suture strangulation of a section of ischemic jejunum with steatitis that presumably occurred during ligation of the left ovarian pedicle 48 h prior. Furthermore, the normal appearance of the urinary tract and the patient's concurrent severe dehydration and hypovolemia supported a prerenal cause for the marked azotemia. Therefore, exploratory laparotomy with resection and anastomosis of the devitalized intestine was recommended to resolve the obstruction along with a reasonable prognosis for renal recovery once perfusion was restored.

**Figure 3:**
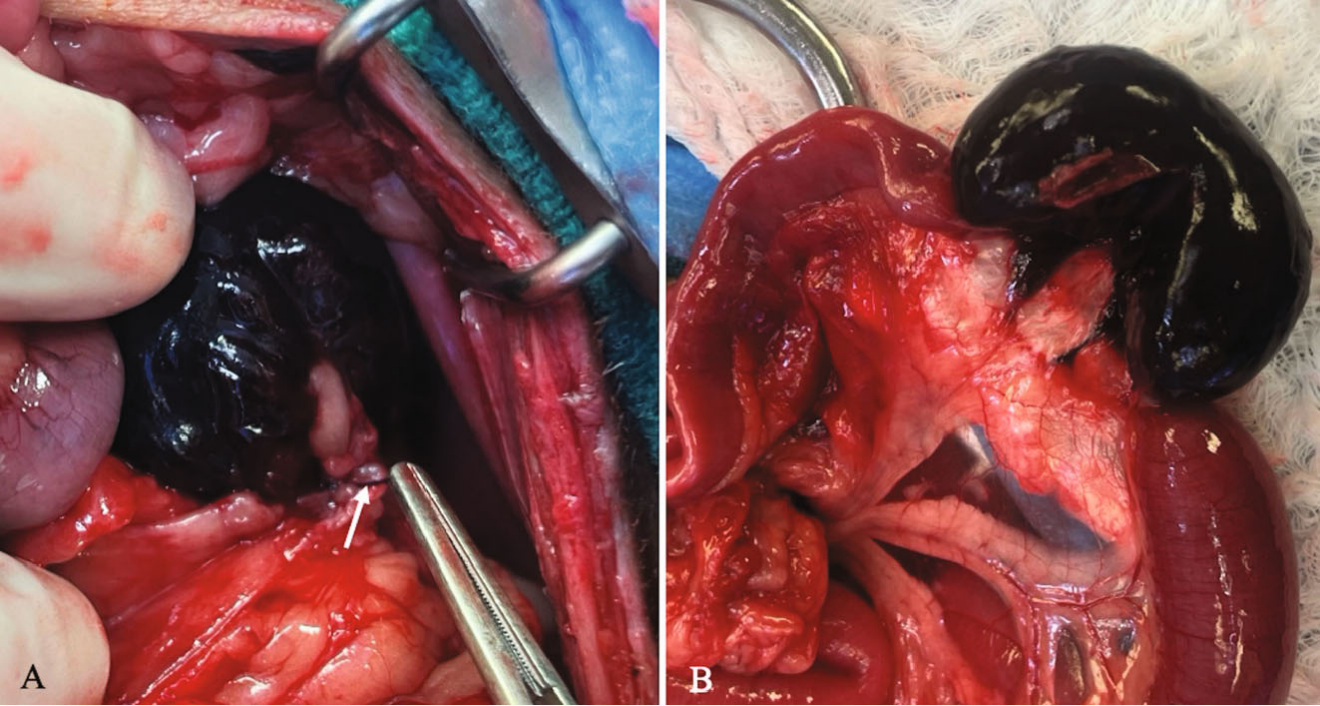
Intraoperative image of the affected jejunal segment strangulated by a ligature (white arrow), that is grasped with Debakey tissue forceps, at the level of the left ovarian pedicle (A). Intraoperative image of the affected jejunal segment, with dilation of the orad jejunum, after removal of the encircling ligature (B).

At anesthetic induction, noninvasive blood pressure was unable to be obtained and rectal temperature was 93°F. Hypotension was treated with IV boluses of LRS (10 mL/kg) and hypertonic saline (4 mL/kg) over 15 min (each) and titrating of isoflurane and norepinephrine CRI (0.2–1 mg/kg/min). Aggressive heat support was instituted prior to induction and continued through recovery with a combination of HotDog (Augustine Surgical Inc., Eden Prairie, Minnesota), Bair Hugger (3M, Saint Paul, Minnesota), and T/Pump warming system (Stryker, Portage, Michigan). Exploratory laparotomy was performed from the xiphoid to the caudal abdomen, extending the previous 7-mm linea incision. Undamaged ureters were identified bilaterally and traced from the urinary bladder to renal pelvises. At the level of the left ovarian pedicle, a 5-cm section of jejunum was dark purple and entrapped in a suture ligature (Figure 3). The ligature was removed and a jejunal resection and simple continuous handsewn anastomosis was performed using a 4-0 polydioxanone suture. The dog recovered uneventfully from anesthesia and the creatinine normalized the following morning (1.2 mg/dL) with a persistently increased BUN (78 mg/dL). The dog was initially fed via the nasogastric tube before eating on her own 2 days postoperatively, and at that time, her azotemia had resolved (creatinine 0.54 mg/dL, BUN 26 mg/dL). She was discharged the following day and reported to be doing well at follow-up 1 month later.

## 3. Discussion

To the author's knowledge, intestinal ligation post-OVH has not been previously reported in dogs. Other differentials considered at the time of dog's presentation include ureteral injury and acute kidney injury (AKI) related to general anesthesia or meloxicam.

The most common differential for acute azotemia immediately post-OVH is ureteral injury due to their proximity to the ovarian pedicles, as a ureter can be entrapped in an encircling ligature around the ovarian pedicle [1]. In humans, iatrogenic ureteral injury is most commonly reported in gynecologic, intrapelvic colorectal, and urologic procedures, but these events are still rare occurring in < 1% of cases [8]. While the incidence of ureteral ligation in veterinary medicine has not been reported, a single retrospective study described ureteral injuries during OVH and reported that cats were overrepresented compared to dogs. [5] Similar to the chihuahua described in this report, cats may be more likely to have a ureter ligated due to their small size. In addition to this retrospective study, numerous case reports have described iatrogenic ureteral injury post-OVH as well as a variety of surgical correction techniques [9–12]. The most common clinical signs after ureteral ligation include anorexia, vomiting, and lethargy shortly after OVH, which were the presenting complaints in this case [5].

Anesthetic-related AKI was considered a less likely differential given the lack of complications reported in the routine anesthesia log; however, failure to properly monitor for or report hypotension could not be completely ruled out. Meloxicam-induced AKI was also considered less likely as only a single, appropriate dose was given. While nonsteroidal anti-inflammatory (NSAID) drugs can induce idiosyncratic reactions, these are primarily hepatopathies while renal injury generally requires cumulative doses [13]. A single dose of an NSAID can reduce renal blood flow via prostaglandin inhibition, which could have compounded with the hypovolemia due to gastrointestinal losses to induce renal hypoperfusion [14]. Lastly, in the face of renal disease, it is expected that urine concentrating ability is reduced; however, this dog's urine was relatively concentrated (USG 1.020). These findings in the face of hypovolemia and the rapid resolution of azotemia with appropriate fluid therapy once gastrointestinal losses were controlled support a predominantly prerenal azotemia or “low flow” functional AKI [15]. Yet, a component of renal azotemia cannot be ruled out.

Point-of-care ultrasound and abdominal radiographs revealing severe intestinal fluid distension in the absence of overt renomegaly or hydronephrosis were important initial diagnostics in shifting focus toward intestinal mechanical obstruction. A complete abdominal ultrasound was the key diagnostic in confirming the source of the obstruction by visualizing a hyperechoic band compressing a section of jejunum that formed a severely dilated loop. Doppler flow was used to determine that the affected intestinal segment had no blood flow. Abdominal ultrasound also confirmed that the renal pelvises and ureters were not dilated and that both ureters were intact and tapered appropriately. The use of ultrasound in this case is supported by previous literature, as ultrasound was used in the diagnosis of 84% of ureteral injuries that occurred during OVH [5] and ultrasound has greater accuracy than radiographs in diagnosing intestinal obstruction [16]. These imaging findings provided the indication for emergency exploratory laparotomy and allowed for appropriate counselling of the owners on the associated risks of jejunal resection and anastomosis as well as the good prognosis for renal functional recovery.

The ultrasound findings described here also share similarities to those previously reported in cases of intestinal entrapment secondary to adhesions after OVH [17]. While adhesion formation is a common complication of abdominal surgery in human medicine occurring in 70%–90% of patients, this is less commonly reported in veterinary medicine, with a only a small number of case reports describing adhesions leading to small intestinal or colonic obstruction post-OVH [17–19]. However, unlike intestinal ligation which induced acute life-threatening hypovolemia and intestinal necrosis in this case, adhesions take several weeks to mature with fibrin deposition and result in a delay of clinical signs with most humans experiencing adhesion-induced obstruction up to a month after abdominal surgery [18].

Prior to this case report, there are only two case reports of acute intestinal injury post-OVH and both occurred in young cats [6, 7]. These reports and those of ureteral injury [5] all implicate small size of both the patient and the surgical incision as risk factors for iatrogenic visceral injury during routine OVH. These factors were likely contributed to the intestinal ligation in the present case given the dog's minute size (2 kg) and small linea alba incision from the initial OVH. Thus, as previously recommended, proper surgical visualization, especially in small patients, should be emphasized [5]. This includes making larger incisions, optimizing surgical lighting, utilizing surgical assistants and retractors, and voiding the urinary bladder before surgery.

Limitations of this case report include the inability to explicitly determine the role of factors such as anesthetic complications and NSAID administration with subsequent hypovolemia which may have had on the dog's azotemia. Without serial testing of biomarkers of renal injury, definitive differentiation between prerenal and renal azotemia remains challenging and prolonged prerenal insults inevitably result in renal injury if not corrected rapidly [15]. In addition to the speculated risk factors of OVH complications described above, it is not possible to report on all the possible circumstances that could have contributed to this complication.

## Data Availability

Data sharing is not applicable to this article as no datasets were generated or analyzed during the case report.

## Supporting Information

Supporting Information Additional supporting information can be found online in the Supporting Information section. Figure S1. Cineloop of a jejunal segment in the left mid abdomen and caudal to the left kidney of an 11-month-old, female castrated chihuahua referred for evaluation of severe azotemia post-OVH. Similar to Figure 2, hyperechoic, well-defined suture material encircles a folded and severely dilated jejunal segment forming a hairpin loop. The wall of the affected segment is moderately thickened with maintenance of wall layer distinction and lack of blood flow on microvascular Doppler interrogation, consistent with intestinal necrosis. Marked focal steatitis and mild anechoic peritoneal effusion surround the affected segment.
